# Supplementary material for: How do perceptions of Covid-19 risk impact pregnancy-related health decisions? A convergent parallel mixed-methods study protocol
Source: PLoS One. 2023 Aug 10;18(8):e0288952. doi: 10.1371/journal.pone.0288952 (PMC10414672; doi:10.1371/journal.pone.0288952)
Supplement: S1 Table — (DOCX) [file pone.0288952.s001.docx]

S1 Table. Description of health administrative data sources

| **Dataset** | **Description** | **Intended Purpose** | **Data Holding** | |
| --- | --- | --- | --- | --- |
|  |  |  | **ICES** | **PopDataBC** |
| Ontario Mother-Baby linked dataset (MOMBABY) | MOMBABY includes all inpatient birth admission records from the Discharge Abstract Database. It links mothers and their newborns deterministically based on the maternal/newborn chart number. | - Cohort creation - Baseline characteristics (e.g., parity) - Covariates and confounders of interest (e.g., use of assistive fertility) | X |  |
| British Columbia Perinatal Data Registry (BCPDR) | The PDR contains data abstracted from obstetrical and neonatal medical records on nearly all births in BC from over 60 hospitals as well as births occurring at home attended by registered midwives. | - Cohort creation - Baseline characteristics (e.g., maternal age) - Covariates and confounders of interest (e.g., number of antenatal visits) |  | X |
| Ontario Health Insurance Policy (OHIP) | The OHIP claims database contains information on publicly funded services, primarily provided by physicians, to Ontario residents eligible for the health insurance system. The main data elements are service/billing codes for the service provided. | - Outcome definition:   - Tdap vaccination record - Covariates and confounders of interest (e.g., perinatal care provider type) | X |  |
| Medical Services Plan dataset (MSP) | The MSP dataset includes medically necessary services provided by fee-for-service practitioners to individuals covered by BC's universal insurance program. | - Outcome definition:   - Gestational diabetes screening - Covariates and confounders of interest (e.g., prenatal care visits) |  | X |
| COVaxON | COVaxON is a central data repository for COVID-19 vaccine data and reporting in Ontario. | - Outcome definition:   - COVID-19 vaccination record | X |  |
| COVID-19 Immunization Data | This dataset reports vaccination coverage in BC. | - Outcome definition   - COVID-19 vaccination record |  | X |
| Ontario Laboratory Information System (OLIS) | OLIS provides lab results of patients from all public Health Ontario laboratories, including hospitals and community laboratories. | - Outcome definition:   - Gestational diabetes screening | X |  |
| Discharge Abstract Data  (DAD) | The DAD is a national database that captures administrative, clinical and demographic information on all hospital discharges and day surgeries in Canada. | - Outcome definition:   - Postpartum length-of-stay   - New onset of depression, anxiety or adjustment disorder - Covariates and confounders of interest (e.g., assisted births) | X | X |
| Ontario Mental Health Reporting System  (OMHRS) | OMHRS reports information about individuals who receive adult mental health services in Ontario. | - Outcome definition:   - New onset of depression, anxiety or adjustment disorder | X |  |
| National Ambulatory Care Reporting System (NACRS) | NACRS contains national data for hospital-based and community-based ambulatory care, including emergency departments. | - Covariates and confounders of interest (e.g., emergency department visits for mental health) | X | X |
| Canadian Index of Multiple Deprivation (CIMD) | The CIMD is a geographically based index developed to quantify the degree of marginalization occurring across the country using census data. | - Stratification (e.g., economic dependency) - Covariates and confounders of interest (e.g., ethnocultural composition) | X | X |
| Registered Persons Database (RPDB) | The RPDB contains basic demographic information about anyone who has ever received an Ontario health card number. | - Baseline characteristics (e.g., maternal age) - Covariates and confounders of interest (e.g., rurality) | X |  |
| BC Vital Events and Statistics Births dataset | This dataset includes information about all births registered in the province of BC. | - Baseline characteristics (e.g., marital status of mother) - Covariates and confounders of interest (e.g., birth weight) |  | X |
| Ontario Diabetes Database (ODD) | ODD is a validated registry of all people in Ontario diagnosed with diabetes. | - Baseline characteristics (e.g., pre-existing diabetes) | X |  |
| BC Chronic Disease Registry (BCCDR) | The BCCDR is derived from administrative data sources to identify cases of various chronic diseases. | - Outcome definition:   - New onset of depression, anxiety or adjustment disorder - Baseline characteristics (e.g., incidence flag of diabetes) |  | X |
