# Supplementary material for: How do perceptions of Covid-19 risk impact pregnancy-related health decisions? A convergent parallel mixed-methods study protocol
Source: PLoS One. 2023 Aug 10;18(8):e0288952. doi: 10.1371/journal.pone.0288952 (PMC10414672; doi:10.1371/journal.pone.0288952)
Supplement: S2 Table — (DOCX) [file pone.0288952.s002.docx]

S2 Table. Sample size calculations

**Disclaimer:** Sample size computations are based on anticipated incidence rates for outcomes of interest. However, the incidence rates of our outcomes in the exposure groups (i.e., 2021 and 2022 birth groups) are largely unknown based on current evidence. Our study aims to address this knowledge gap by reporting incidence rates using population-based data and comparing them across birth groups.

Therefore, while the anticipated incidence of our outcomes in the reference groups (i.e., the 2019 birth group and 2022 general non-pregnant population) are informed by recent evidence, the incidence rates for the exposure groups (i.e., 2021 and 2022 birth groups) are estimated based on our hypotheses. The anticipated changes (e.g., -2.5%, -5%) detailed below were arbitrarily chosen to represent minor and moderate changes that align with our hypotheses where evidence was not available. We underestimated the anticipated changes in these incidence rates to overestimate the minimum sample sizes to ensure our study will be adequately powered.

In future papers where we report the findings from this study, we will compute post-hoc power calculations to demonstrate the adequacy of our large, population-based sample in detecting differences in the outcome rates across our groups of interest.

Our calculations were computed using the resource:

Kane SP. Sample Size Calculator. ClinCalc: https://clincalc.com/stats/SampleSize.aspx. Updated July 24, 2019. Accessed April 5, 2023.

Type I/II Error Rate

Alpha = 0.05

Power = 80%

**Outcome 1 – Tdap vaccination**

Statistical Parameters

Anticipated incidence of Tdap vaccination in 2019 birth group: 29.2%

- Source: Fakhraei, R., Fung, S. G., Petrcich, W., Crowcroft, N., Bolotin, S., Gaudet, L., ... & Fell, D. B. (2022). Trends and characteristics of Tdap vaccination during pregnancy in Ontario, Canada: a retrospective cohort study. Canadian Medical Association Open Access Journal, 10(4), E1017-E1026.

Anticipated incidence of Tdap vaccination in 2021 birth group: 26.7%

- Anticipating a minor drop (-2.5%) in vaccination rates as a result of the Covid-19 pandemic

Anticipated incidence of Tdap vaccination in 2022 birth group: 24.2%

- Anticipating a further drop (-5%) in vaccination rates as a result of the Covid-19 pandemic and Covid-19 vaccination efforts

Minimum Sample Size

- For 2019 vs. 2021 birth groups comparison: 5,057 subjects in either group (n=10,114)
- For 2019 vs. 2022 birth groups comparison: 1,228 subjects in either group (n=2,456)
- For 2021 vs. 2022 birth groups comparison: 4,764 subjects in either group (n=9,528)

**Outcome 2 – Covid-19 dose 1 vaccination**

Statistical Parameters

Anticipated incidence of Covid-19 dose 1 vaccination in 2022 birth group: 74.6%

- Source: BORN Ontario. (2023). Covid-19 vaccination during pregnancy in Ontario. Technical report. Accessed on April 6, 2023: https://www.bornontario.ca/en/whats-happening/resources/Documents/Report_2.pdf

Anticipated incidence of Covid-19 dose 1 vaccination in general (non-pregnant) female population: 89.5%

- Source: Public Health Ontario. (2023). Ontario Covid-19 data tool.

Minimum Sample Size

- 103 subjects in either group (n=206)

**Outcome 3 – Covid-19 dose 2 vaccination**

Statistical Parameters

Anticipated incidence of Covid-19 dose 2 vaccination in 2022 birth group: 75.3%

- Source: BORN Ontario. (2023). Covid-19 vaccination during pregnancy in Ontario. Technical report. Accessed on April 6, 2023: https://www.bornontario.ca/en/whats-happening/resources/Documents/Report_2.pdf

Anticipated incidence of Covid-19 dose 2 vaccination in general (non-pregnant) female population: 87.0%

- Source: Public Health Ontario. (2023). Ontario Covid-19 data tool.

Minimum Sample Size

- 174 subjects in either group (n=348)

**Outcome 4 – GDM screening**

Statistical Parameters

Anticipated incidence of gestational diabetes screening in 2019 birth group: 95.5%

- Source: Fakhraei, R., Fung, S. G., Petrcich, W., Crowcroft, N., Bolotin, S., Gaudet, L., ... & Fell, D. B. (2022). Trends and characteristics of Tdap vaccination during pregnancy in Ontario, Canada: a retrospective cohort study. Canadian Medical Association Open Access Journal, 10(4), E1017-E1026.

Anticipated incidence of gestational diabetes screening in 2021 birth group: 93.0%

- Anticipating a minor drop (-2.5%) in screening rates as a result of the Covid-19 pandemic

Anticipated incidence of gestational diabetes screening in 2022 birth group: 95.5%

- Anticipating a similar incidence of gestational diabetes screening compared to 2019 birth group

Minimum Sample Size

- For 2019 vs. 2021 birth groups comparison: 1,360 subjects in either group (n=2,720)
- For 2019 vs. 2022 birth groups comparison: 1,360 subjects in either group (n=2,720)

**Outcome 5 – Postpartum length-of-stay**

Statistical Parameters

Anticipated mean(SD) of postpartum length-of-stay in 2019 birth group: 2.5(1.8) days

- Source: Public Health Agency of Canada. (2008). Canadian perinatal health report. Technical report. Accessed on April 6, 2023: https://publications.gc.ca/collections/collection_2009/aspc-phac/HP10-12-2008E.pdf

Anticipated decrease in postpartum length-of-stay in 2021 birth group: -5%

- Anticipating a moderate drop (-5%) in length-of-stay as a result of the Covid-19 pandemic

Anticipated incidence of gestational diabetes screening in 2022 birth group: -2.5%

- Anticipating a minor drop (-2.5%) in length-of-stay as a result of the Covid-19 pandemic

Minimum Sample Size

- For 2019 vs. 2021 birth groups comparison: 3,532 subjects in either group (n=7,064)
- For 2019 vs. 2022 birth groups comparison: 14,128 subjects in either group (n=28,256)

**Outcome 6 – New mental health diagnosis during pregnancy**

Statistical Parameters

Anticipated incidence of onset of depression, anxiety, or adjustment disorder during pregnancy in 2019 birth group: 15%

- Source: The Centre for Addiction and Mental Health. (2023). Perinatal mood and anxiety disorders. Website. Accessed on April 6, 2023: https://www.camh.ca/en/professionals/treating-conditions-and-disorders/perinatal-mood-and-anxiety-disorders

Anticipated incidence of depression, anxiety, or adjustment disorder during pregnancy in 2021 birth group: 20%

- Anticipating a moderate increase (5%) in mental health diagnoses as a result of the Covid-19 pandemic

Anticipated incidence of depression, anxiety, or adjustment disorder during pregnancy in 2022 birth group: 20%

- Anticipating a moderate increase (5%) in mental health diagnoses as a result of the Covid-19 pandemic

Minimum Sample Size

- For 2019 vs. 2021 birth groups comparison: 905 subjects in either group (n=1,810)
- For 2019 vs. 2022 birth groups comparison: 905 subjects in either group (n=1,810)

**Outcome 7 – New mental health diagnosis postpartum**

Statistical Parameters

Anticipated incidence of postpartum depression, anxiety, or adjustment disorder in 2019 birth group: 7.5%

- Source: Public Health Agency of Canada. (2016). Pregnancy and women’s mental health in Canada. Technical report. Accessed on April 6, 2023: https://health.canada.ca/publications/healthy-living-vie-saine/pregnancy-mental-health-grossesse-sante-mentale/alt/pregnancy-mental-health-grossesse-sante-mentale-eng.pdf

Anticipated incidence of postpartum depression, anxiety, or adjustment disorder in 2021 birth group: 12.5%

- Anticipating a moderate increase (5%) in mental health diagnoses as a result of the Covid-19 pandemic

Anticipated incidence of postpartum depression, anxiety, or adjustment disorder in 2022 birth group: 12.5%

- Anticipating a moderate increase (5%) in mental health diagnoses as a result of the Covid-19 pandemic

Minimum Sample Size

- For 2019 vs. 2021 birth groups comparison: 564 subjects in either group (n=1,128)
- For 2019 vs. 2022 birth groups comparison: 564 subjects in either group (n=1,128)
